# Systematic review and meta-analysis of oxidative stress and antioxidant markers in recurrent aphthous stomatitis

**DOI:** 10.1186/s12903-023-03636-1

**Published:** 2023-12-02

**Authors:** Saeideh Ghasemi, Fataneh Farokhpour, Bardia Mortezagholi, Emad Movahed, Arshin Ghaedi, Morad Kohandel Gargari, Monireh Khanzadeh, Aida Bazrgar, Shokoufeh Khanzadeh

**Affiliations:** 1 Dental school, Tabriz University of Medical Sciences, Tabriz, Iran; 2 Department of pathology, school of medicine, Isfahan university of medical science, Isfahan, Iran; 3 Dental Research Center, Faculty of Dentistry, Islamic Azad University of Medical Sciences, Tehran, Iran; 4 Student Research Committee, School of Medicine, Shiraz University of Medical Sciences, Shiraz, Iran; 5 Tabriz University of Medical Sciences, Tabriz, Iran; 6 Geriatric & Gerontology Department, Medical School, Tehran University of medical and health sciences, Tehran, Iran

**Keywords:** Stomatitis, Aphthous, oxidative stress, Meta-analysis

## Abstract

**Background:**

We performed this systematic review and meta-analysis to synthesize all studies that reported the level of oxidative and antioxidative markers in recurrent aphthous stomatitis (RAS) patients compared to controls.

**Methods:**

We registered our study in PROSPERO (CRD42023431310). PubMed, ProQuest, Scopus, EMBASE, Google Scholar, and Web of Science were searched to find relevant publications up to June 5, 2023. The standardized mean differences (SMDs) with 95% confidence intervals (CIs) were calculated. We included 30 articles after multiple stags of screening.

**Results:**

We found that erythrocyte superoxide dismutase and Glutathione peroxidase activity were significantly lower in patients with RAS compared to healthy controls (SMD = − 1.00, 95%CI = -1.79 to -0.21, *p* = 0.013, and SMD = − 1.90, 95%CI = -3.43 to -0.38, *p* = 0.01, Respectively). However, there was not any difference between patients with RAS and healthy controls in erythrocyte Catalase (SMD = − 0.71, 95%CI = -1.56–0.14, *p* = 0.10). The total antioxidant status (TAS) level, in serum was significantly lower in patients than healthy controls (SMD = − 0.98, 95%CI = -1.57 to -0.39, *p* = 0.001). In addition, RAS patients had higher levels of serum Malondialdehyde (MDA), Serum total oxidant status, and serum oxidative stress index than healthy controls (SMD = 2.11, 95%CI = 1.43–2.79, *p* < 0.001, SMD = 1.53, 95%CI = 0.34–2.72, *p* = 0.01, and SMD = 1.25, 95%CI = 0.25–2.25, *p* = 0.014, Respectively); However, salivary MDA and TAS, and serum uric acid, vitamin E and C, and reduced glutathione levels of patients with RAS were not different from that of healthy controls.

**Conclusions:**

The relationship between oxidative stress and RAS is well established in this meta-analysis. Although the molecular processes underlying the etiology of this pathology remain unknown, evidence indicating oxidative stress has a significant role in the pathogenesis of RAS has been revealed.

**Supplementary Information:**

The online version contains supplementary material available at 10.1186/s12903-023-03636-1.

## Introduction

A chronic, recurrent, idiopathic inflammatory illness of the oral mucosa is known as recurrent aphthous stomatitis (RAS), which is described as variable degrees of painful ulcers. RAS is a condition that affects over 25% of the world’s population and is widespread [1, 2]. RAS lesions’ development is influenced by bacterial or viral infections, trauma, stress, malnutrition, systemic illnesses, immune system conditions, or genetic predispositions [3, 4]. These factors can cause systemic inflammation, harming the balance between oxidants and antioxidants. Also, oxidative stress and RAS have been linked [1, 3–5].

In order to cleanse the radicals produced by the generation of reactive oxygen species (ROS), the body develops antioxidant systems; however, oxidative stress induces an imbalance in these antioxidant mechanisms. Antioxidants regulate ROS production under normal physiological circumstances so that it does not affect the tissues and organs [6, 7]. The balance shifts in favor of the oxidants when the ROS concentration surpasses pathological levels, and the exogenous and endogenous antioxidants cannot neutralize these radicals [8]. As a result, components in the body, like lipids, DNA, and proteins, are harmed by oxidation. According to several recent studies, DNA damage and oxidative stress are closely related [9, 10].

When a stressed organism fails to eliminate an excess of endogenous free radicals, these highly reactive chemicals irreversibly damage cell structures and generate mutations in various illnesses’ etiology. Oxidative stress accelerates the aging process and contributes to the development of degenerative and chronic illnesses [7, 11]. The progression of the pathway activated by free radical activity is often obscured and only becomes evident when the clinical presentation is already severe. Arthritis, cancer, cataract, autoimmune diseases, retinitis pigmentosa, neurodegenerative illnesses, and cardiovascular disorders (like heart attack, hypertension, stroke, and atherosclerosis) are all caused by an abnormal generation of free radicals [12–14]. Furthermore, the relationship between oxidative stress and RAS has been studied. For example, superoxide dismutase (SOD), Glutathione peroxidase (GPx), and catalase (CAT) are erythrocyte antioxidant enzymes, playing a crucial role in the oxidative stress defense system and have been linked to RAS [15–44]. In addition, total oxidant status (TOS), uric acid (UA), vitamin E (Vit E), and vitamin C (Vit C), other markers of antioxidant level, were studies in RAS patients. In contrast, Malondialdehyde (MDA), reduced Glutathione (GSH), total oxidant status (TOS), oxidative stress index (O SI) (TOS/ Total antioxidant status (TAS)) levels are markers for oxidative stress in RAS [15–44]. Nevertheless, the findings have remained controversial. Consequently, we planned to perform a systematic review and meta-analysis to synthesize all studies that reported the level of oxidative and antioxidative markers in RAS patients compared to controls in order to recognize whether or not these indicators are viable biomarkers for RAS. This is, to the best of our knowledge, the first meta-analysis on this topic.

Our meta-analysis revised that oxidative stress and antagonistic indicators may be viable biomarkers for RAS diagnosis.

## Materials and methods

This study is performed based on the Preferred Reporting Items for Systematic Reviews and Meta-analyses (PRISMA) 2020 reporting guideline.

### Search strategy

Two independent authors carried out a comprehensive, time-limit-free search of the databases PubMed, ProQuest, Scopus, EMBASE, Google Scholar and Web of Science to find relevant publications that measured oxidant and antioxidant levels (updated June 5, 2023). The search strategy was as follows: (“Oxidant”[All Fields] OR “reactive oxygen”[All Fields] OR “Oxidative”[All Fields] OR “antioxidant”[All Fields] OR “Oxidative”[All Fields] OR “oxidants”[MeSH Terms] OR “antioxidants”[MeSH Terms]) AND (“Recurrent aphthous stomatitis”[All Fields] OR “stomatitis, aphthous”[MeSH Terms]). Several search keywords were utilized to find the relevant literature, and the search strategies were tailored to each database. We registered our study in PROSPERO (CRD42023431310). In addition, supplementary file 1 shows the PRISMA checklist of this study. Inter-reviewer agreement was assessed using kappa statistic [45]. Significant agreement was defined Kappa value of > 0.6.

### Study selection and data extraction

According to the following inclusion criteria, studies were chosen by two independent authors: (1) case-control study, (2) laboratory assessment of oxidant and antioxidant status, (3) random sampling or cluster sampling, (4) human subjects, (5) full article access, and (6) clear diagnostic criteria.

The exclusion criteria were as follows:

1. Studies that do not focus on diagnostic aspects of NLR in RAS patients.
2. Non-peer-reviewed, or unpublished studies.
3. Studies providing odds ratio(OR) or hazard ratio, instead of mean and standard deviation.
4. in-silico, in-vitro, and animal studies.
5. Letters to the editor, reviews, conference abstracts, and other nonclinical literature.

Articles in the full text were assessed for eligibility. The following information was gathered separately by the two reviewers for each study: name of the first author, country, publication year, sample size, mean age, male percentage, percentage of patients with minor RAS, and the mean and standard deviation of oxidative and antioxidantive markers. The authors resolved their disagreements with a discussion. EndNote X7.4 software (London, UK: Clarivate) was used for duplicate removal and screening.

### Quality assessment

The Newcastle-Ottawa Scale (NOS) served as the foundation for the quality evaluations of the studies. Two researchers independently analyzed each study’s quality score and assessed the eight categories (8 items), i.e., selection, comparability, and exposure, for case-control studies. Studies with NOS score ≥ eight were considered high-quality literature. In addition, a score of 7 or 6 was considered as medium quality.

### Statistical analysis

The meta-analysis was executed using Stata 11.2 software (Stata Corp, College Station, TX). The standardized mean differences (SMDs) in TAS, TOS, MDA, and CAT levels between RAS patients and control groups, and 95% confidence intervals (CIs), were calculated for each study. With the use of the I^2^ statistic and χ2-based Q statistic, the between-study heterogeneity was analyzed. We considered I2>75% and P χ2 test < 0.05 as significant heterogeneity of results; In such a case, we used random-effects model; otherwise, we used fixed-effect model. Meta-regression analysis was conduct to find the source of heterogeneity. Subgroup analysis was conducted based on quality score and country in which the study was conducted. Sensitivity analysis was conducted to evaluate the stability of the findings. Egger’s test and a funnel plot analysis were used to examine publication bias. A statistically significant difference between groups was a *P* value < 0.05 (two-tailed).

## Results

### Search results and included studies

**Fig. 1:**
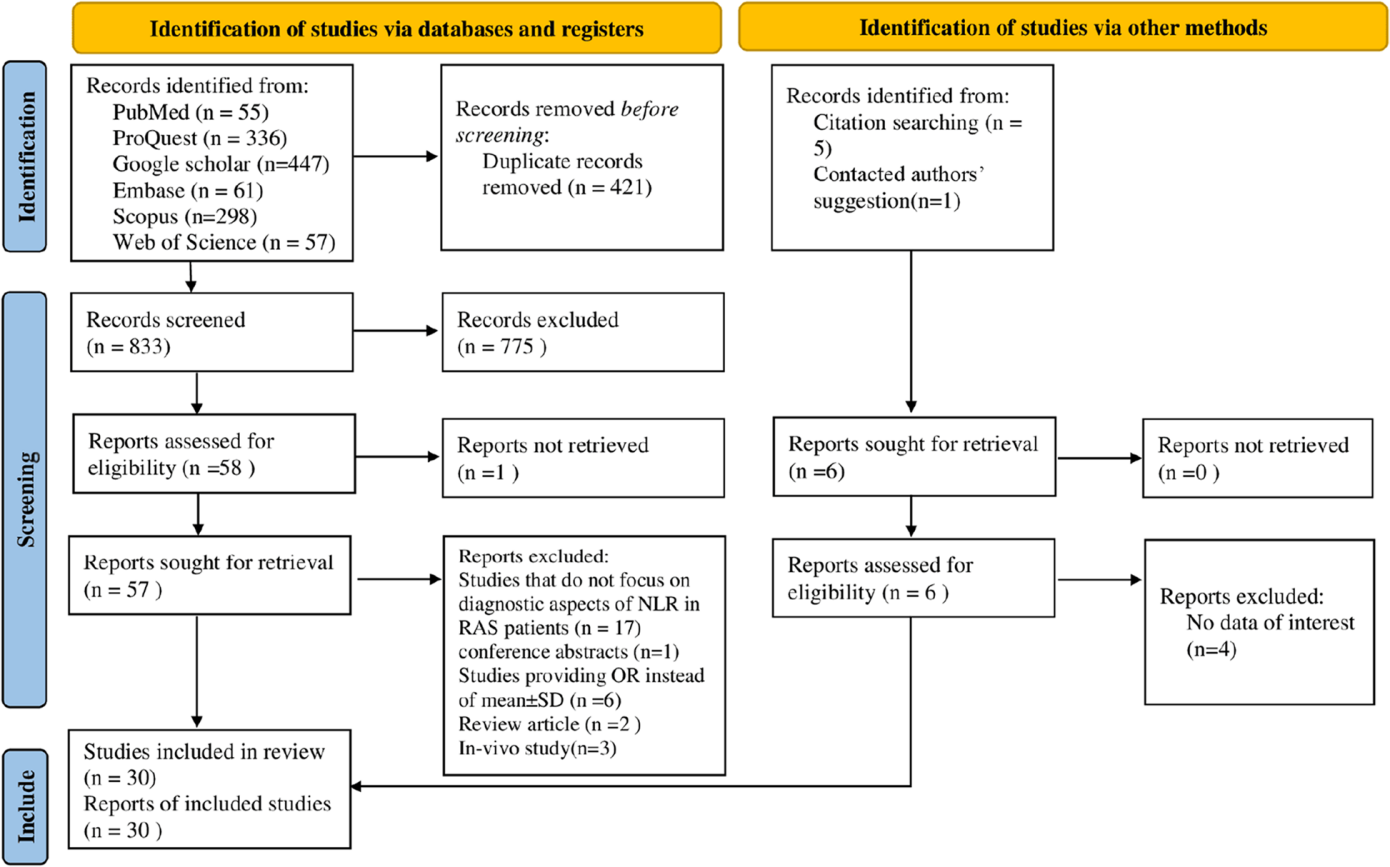
PRISMA 2020 Flow diagram for new systematic reviews which includes searches of databases, registers and other sources

As shown in Fig. 1, we found 1260 articles in the initial search and deleted the duplicates (*n* = 421). Then, two authors (ShKh and AGh) screened the 833 studies based on title and abstract. The remaining articles (*n* = 57) were screened based on their full text. Finally, 30 articles remained for meta-analysis (15–44). A high agreement between the authors screening articles was found (94% agreement; kappa = 0.82; 95%CI = 0.64–1.0, *P* < 0.001).

### Characteristics of the population and quality assessment

In total, 30 articles were included in the analysis, including 1179 patients with RAS and 1147 healthy controls [15–44]. Table 1 shows the overall characteristics and quality scores of the included articles. Supplementary file 2 shows all the original data extracted from studies, and used in the meta-analysis. In addition, supplementary file 3 shows details of quality assessment of included studies.

**Table 1:** Characteristic of included studies

Author, year (Reference number)	Country	Factors assessed	Study design	Mean age	Sex ratio	Minor RAS ratio	NOS score
Arikan,2009 [15]	Turkey	Serum: MDA, GPx, Vit E	Prospective	38.39	38.46	Not declared	8
Cimen, 2003 [16]	Turkey	Serum: MDA, SOD, CAT, GPx,	Prospective	30.04	50	86.36	7
Babaee, 2016 [17]	Iran	Saliva: MDA, TAS	Prospective	34.7	53.6	100	8
Akoglu, 2013 [18]	Turkey	Serum: TAS, TOS, OSI	Prospective	30.5	52.3	100	8
Ziaudeen, 2001 [19]	India	Saliva: MDA, UA Serum: SOD	Prospective	33.13	38.33	Not declared	8
Bilgili, 2013 [20]	Turkey	Serum: TAS, TOS, OSI	Prospective	32	64.51	100	7
Avci, 2014 [21]	Turkey	Serum: MDA, GSH, TAS, TOS, OSI	Prospective	28.9	48	100	7
Khademi, 2014 [22]	Iran	Saliva: MDA, Serum: MDA, Vit E, Vit C	Prospective	29.16	20	Not declared	6
Altinyazar, 2006[23]	Turkey	Serum: MDA, SOD, CAT	Prospective	32.0	53.84	100	7
Caglayan, 2008 [24]	Turkey	Saliva: TAS, Serum: TOS, OSI	Prospective	27.50	48	100	6
Azizi, 2012 [25]	Iran	Saliva: TAS	Prospective	35.6	36	Not declared	6
Saral, 2005 [26]	Turkey	Saliva: MDA, Serum: MDA, Vit E, Vit C	Prospective	35.07	49	Not declared	9
Momen, 2010 [27]	Iran	Saliva: TAS Serum: TAS, SOD, CAT, GPx,	Prospective	28.13	33.33	Not declared	8
Ozturk, 2013 [28]	Turkey	Serum: MDA SOD, CAT, GPx,	Prospective	31.69	33.33	100	8
Gupta, 2014 [29]	India	Serum: SOD, CAT, GPx	Prospective	25.76	56.66	100	8
Ekinci, 2019 [30]	Turkey	Serum: TAS, TOS, OSI	Prospective			Not declared	7
Turgul, 2016 [31]	Turkey	Serum: TAS, TOS, OSI	Prospective	27.14	57.5	100	8
Al-Essa, 2013 [32]	Iraq	Saliva: MDA, TAS Serum: MDA, TAS	Prospective	34.03	46.66	60	6
Li, 2016 [33]	China	Saliva: UA Serum: MDA, Vit E, Vit C	Prospective	31.4	43.29	100	7
Jesija, 2017 [34]	India	Saliva: UA Serum: SOD, CAT, GPx	Prospective	26.2	47.5	70	8
Zhang, 2018 [35]	China	Serum: TAS	Prospective	29.8	42.22	100	7
Sebea, 2020 [36]	Iraq	Saliva: MDA Serum: MDA, SOD, GSH, CAT, UA	Prospective	36.53	33.33	80	8
Rezaei, 2018 [37]	Iran	Saliva: TAS	Prospective	35	57.14	100	9
Bagan, 2014 [38]	Spain	Serum: MDA, GSH	Prospective	41.29	39.3	71.42	8
Zhang, 2017 [39]	China	Serum: SOD, CAT, GPx	Prospective	31.4	43.29	100	7
Hussein, 2016 [40]	Iraq	Serum: MDA,TAS	Prospective	30.1	73.80	100	7
Gunduz, 2004 [41]	Turkey	Serum: SOD, CAT	Prospective	35.07	34.61	75	7
Yardim, 2006 [42]	Turkey	Serum: MDA Saliva: UA	Prospective	40.2	34.78	Not declared	6
Kurku, 2022[43]	Turkey	Serum: TAS,TOS,OSI	Prospective	29.0	42.5	Not declared	7
Zhang, 2022 [44]	China	Serum: GSH	Prospective	30.91	43.67	100	7

*NOS* The Newcastle-Ottawa Quality Assessment Scale, *RAS *Recurrent aphthous stomatitis, *SOD *Superoxide dismutase, *CAT *Catalase, *TOS *Total antioxidant status, *UA *Uric acid, *Vit E *Vitamin E, *Vit C *Vitamin C, *MDA* Malondialdehyde, *OSI *Oxidative stress index, *GSH *Reduced glutathione, *GPx *Glutathione peroxidase, *TAS *Total antioxidant status

### Differences in oxidant and antioxidant level between patients with RAS and healthy controls

**Fig. 2:**
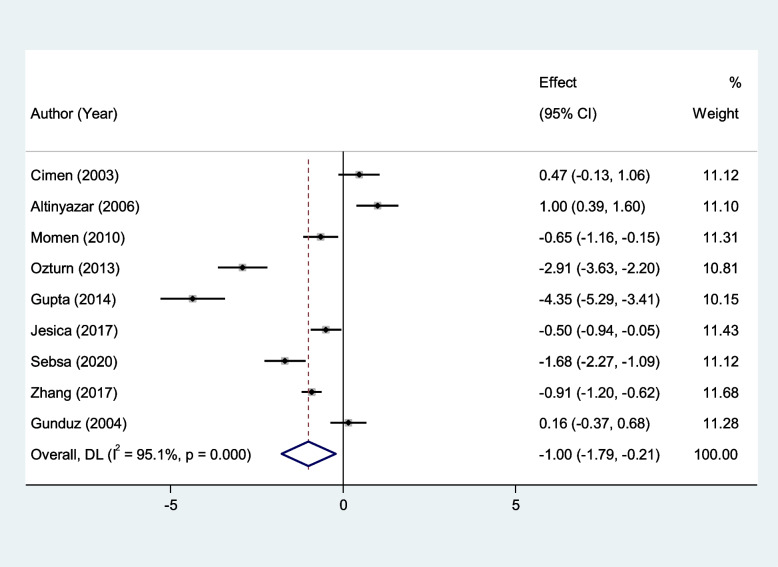
Meta-analysis of differences in erythrocyte superoxide dismutase activity between patients with recurrent aphthous stomatitis and healthy controls

**Fig. 3:**
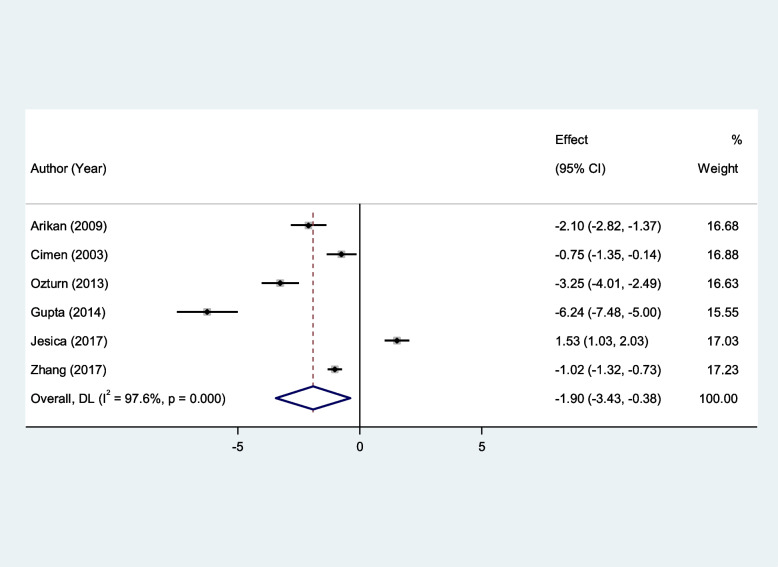
Meta-analysis of differences in erythrocyte Glutathione peroxidase activity between patients with recurrent aphthous stomatitis and healthy controls

**Fig. 4:**
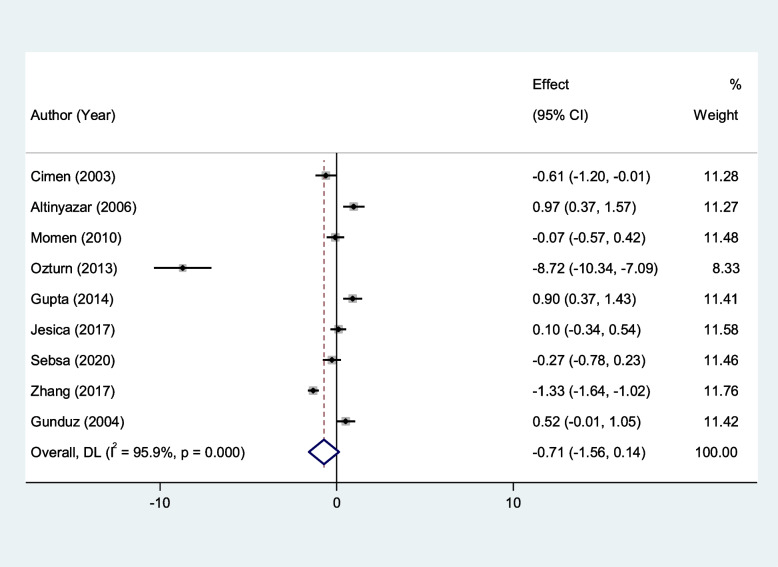
Meta-analysis of differences in erythrocyte Catalase activity between patients with recurrent aphthous stomatitis and healthy controls

We found that erythrocyte SOD and GPx activity were significantly lower in patients with RAS compared to healthy controls (SMD = − 1.00, 95%CI = -1.79 to -0.21, *p* = 0.013, random-effects model, Fig. 2, and SMD = − 1.90, 95%CI = -3.43 to -0.38, *p* = 0.01, random-effects model, Fig. 3, Respectively). However, there was not any significant difference between patients with RAS and healthy controls in erythrocyte CAT (SMD = − 0.71, 95%CI = -1.56–0.14, random-effects model, Fig. 4, *p* = 0.10).

**Fig. 5:**
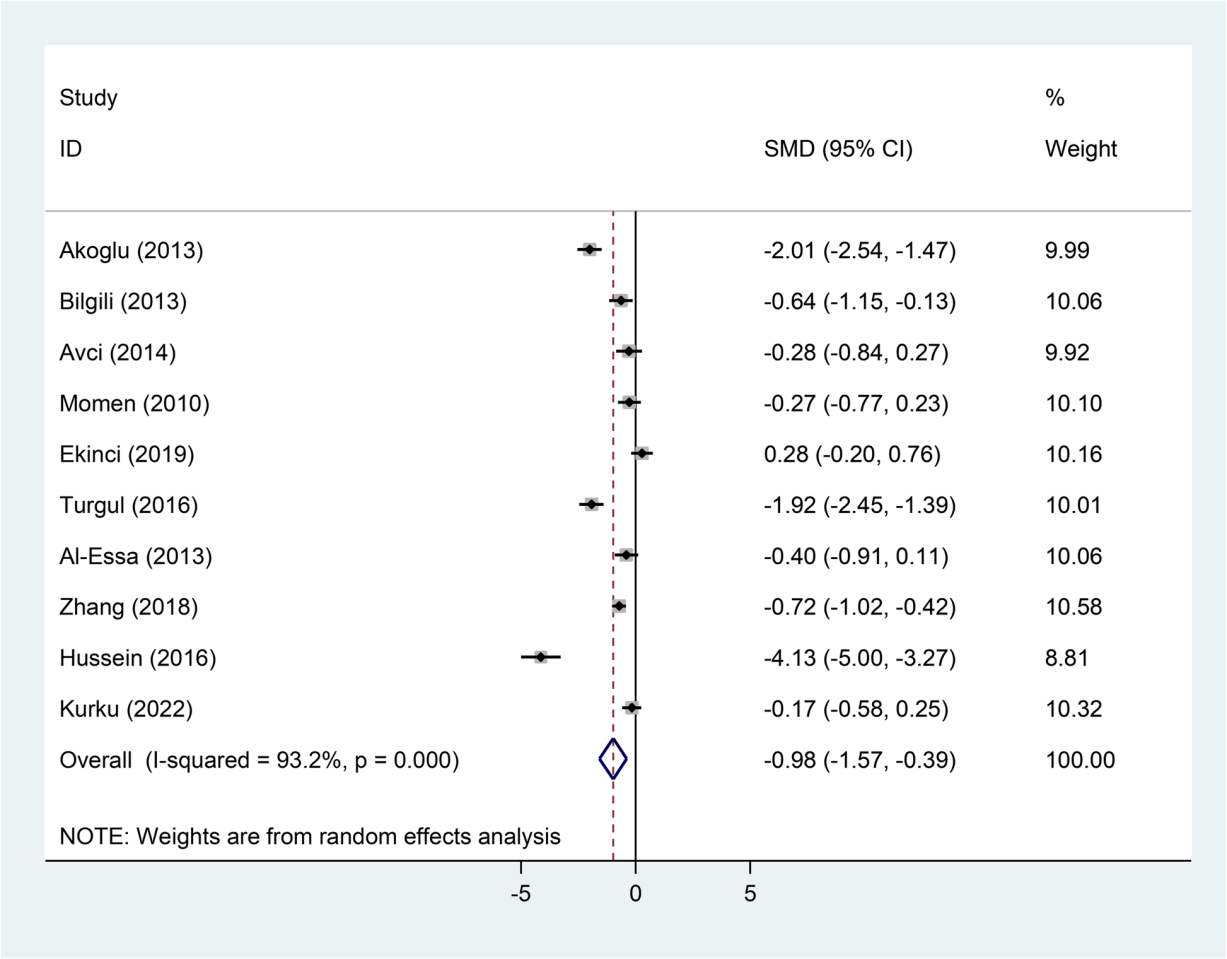
Meta-analysis of differences in serum total antioxidant status level between patients with recurrent aphthous stomatitis and healthy controls

**Fig. 6:**
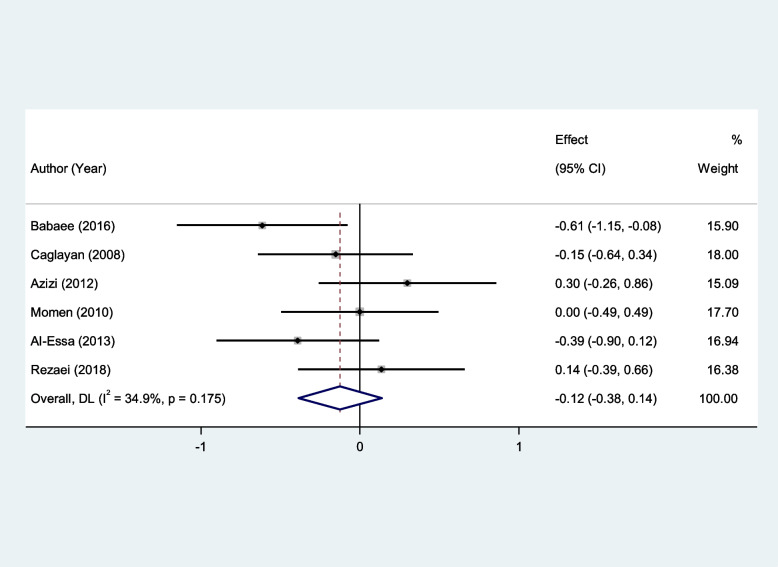
Meta-analysis of differences in salivary total antioxidant status between patients with recurrent aphthous stomatitis and healthy controls

The TAS level in serum was significantly lower in patients than healthy controls (SMD = − 0.98, 95%CI = -1.57 to-0.39, *p* = 0.001, random-effects model, Fig. 5); However, in salivary, there was not any difference (SMD = − 0.12, 95%CI = -0.38–0.14, fixed-effect model, *p* = 0.33, Fig. 6).

**Fig. 7:**
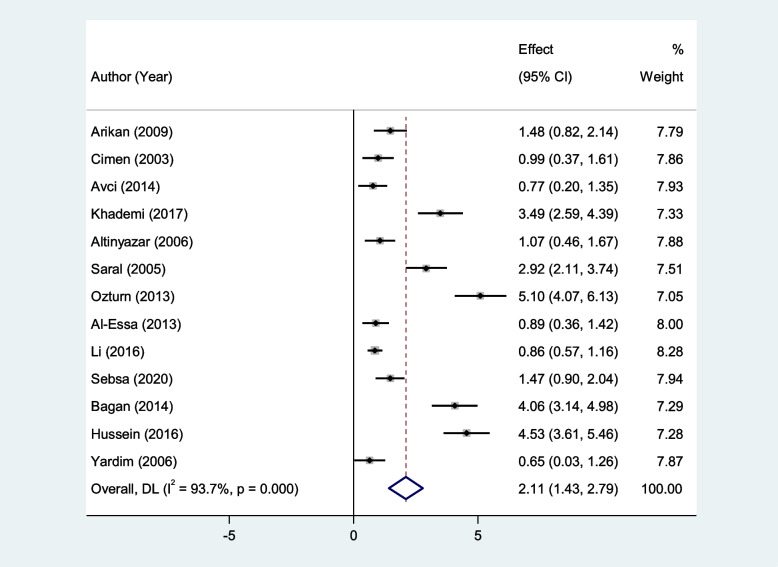
Meta-analysis of differences in serum Malondialdehyde between patients with recurrent aphthous stomatitis and healthy controls

**Fig. 8:**
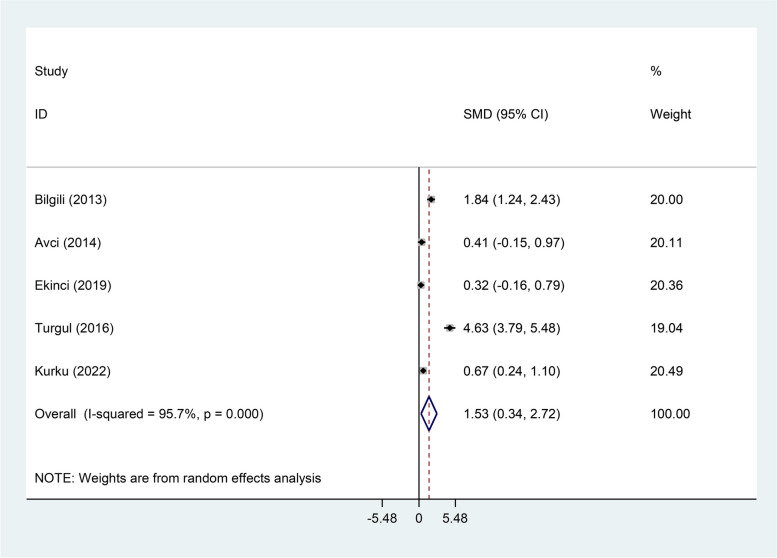
Meta-analysis of differences in serum total oxidant status level between patients with recurrent aphthous stomatitis and healthy controls

**Fig. 9:**
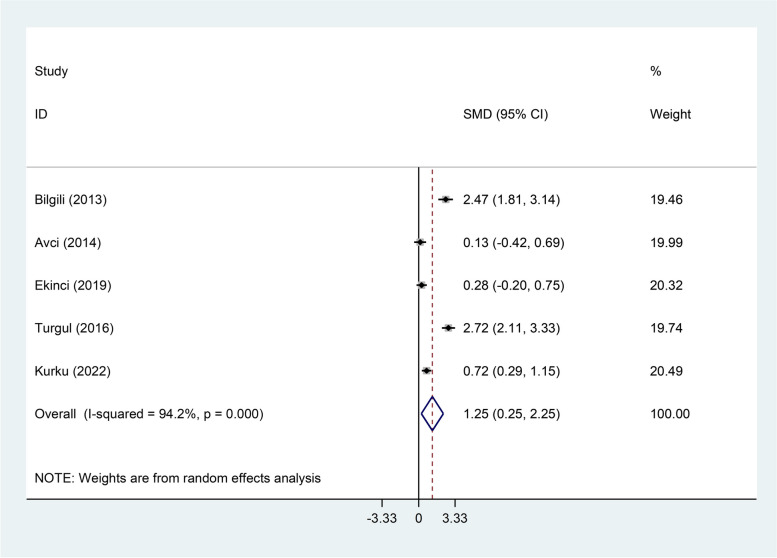
Meta-analysis of differences in serum oxidative stress index level between patients with recurrent aphthous stomatitis and healthy controls

**Fig. 10:**
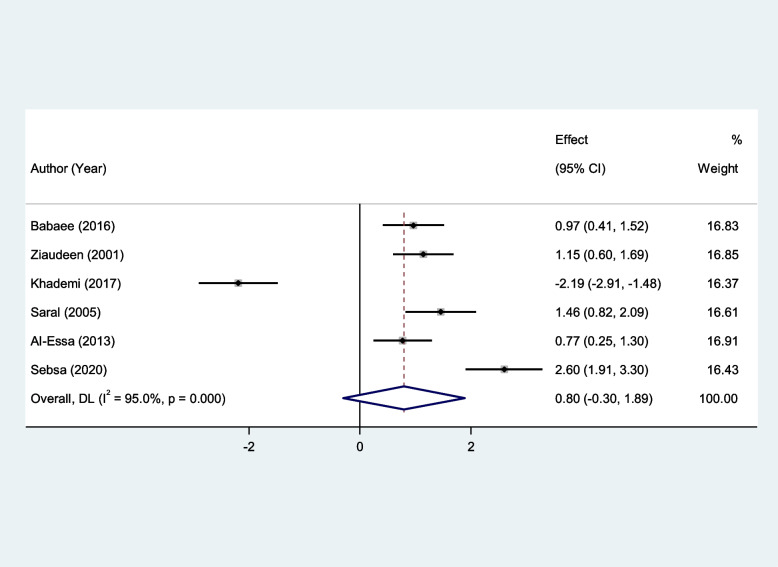
Meta-analysis of differences in salivary Malondialdehyde level between patients with recurrent aphthous stomatitis and healthy controls

RAS patients had significantly higher levels of serum MDA, Serum TOS, and serum OSI than healthy controls (SMD = 2.11, 95%CI = 1.43–2.79, *p* < 0.001, random-effects model, Fig. 7, SMD = 1.53, 95%CI = 0.34–2.72, *p* = 0.01, random-effects model, Fig. 8, and SMD = 1.25, 95%CI = 0.25–2.25, *p* = 0.014, random-effects model, Fig. 9, Respectively); However, there was not any significant difference between patients with RAS and healthy controls in salivary MDA (SMD = 0.80, 95%CI = -0.30–1.89, *p* = 0.15, random-effects model, Fig. 10).

**Fig. 11:**
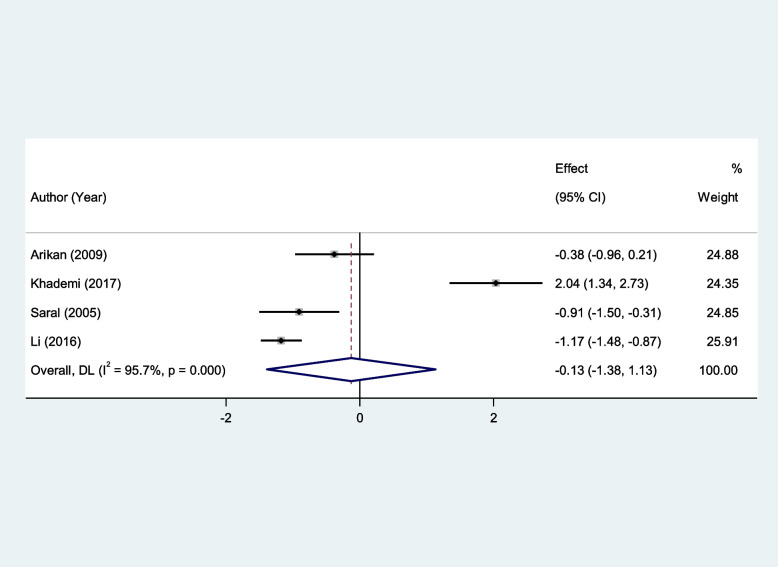
Meta-analysis of differences in serum vitamin E level between patients with recurrent aphthous stomatitis and healthy controls

**Fig. 12:**
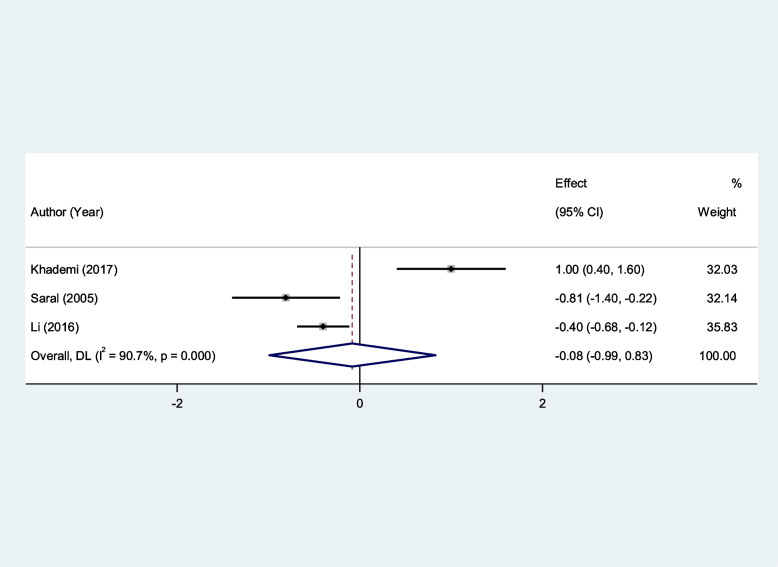
Meta-analysis of differences in serum vitamin C level between patients with recurrent aphthous stomatitis and healthy controls

In addition, serum Vit E and serum Vit C levels of patients with RAS were not significantly different from that of healthy controls (SMD = − 0.13, 95%CI = -1.38–1.13, *p* = 0.84, random-effects model, Fig. 11, and SMD = − 0.08, 95%CI = -0.99–0.83, *p* = 0.85, random-effects model, Fig. 12).

**Fig. 13:**
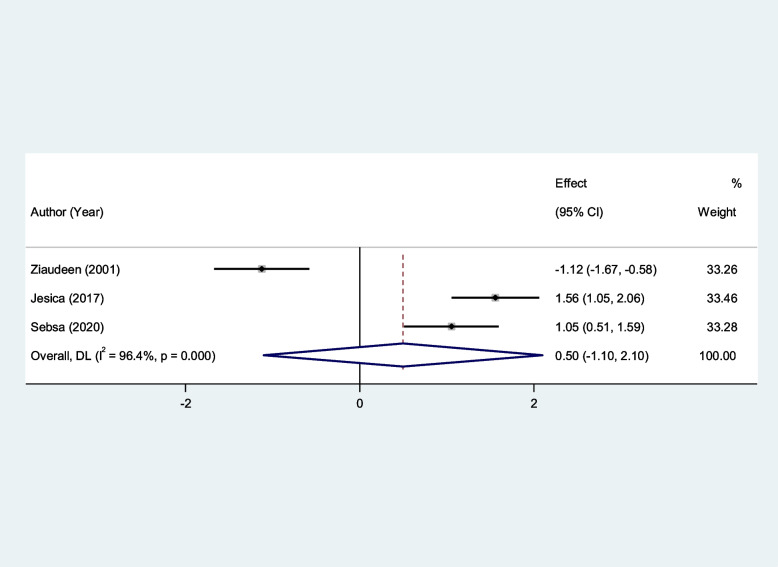
Meta-analysis of differences in salivary uric acid level between patients with recurrent aphthous stomatitis and healthy controls

**Fig. 14:**
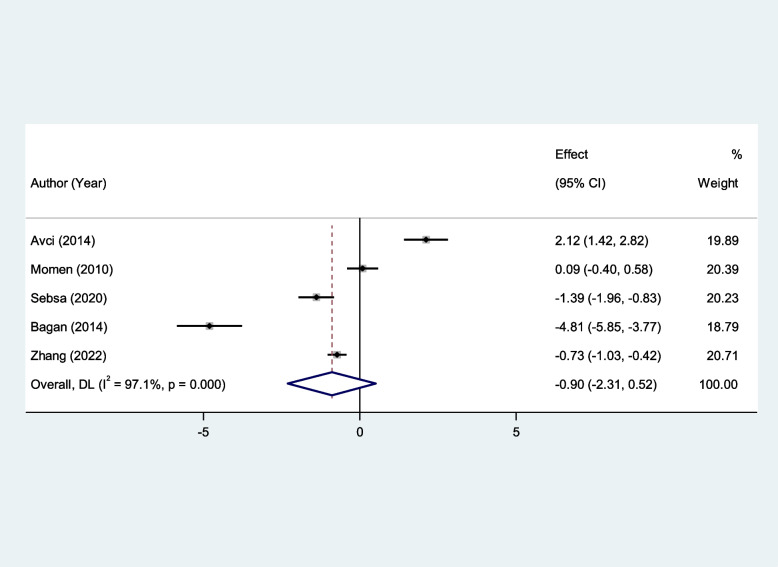
Meta-analysis of differences in serum reduced glutathione level between patients with recurrent aphthous stomatitis and healthy controls

Similarly, salivary UA and serum GSH levels of patients with RAS were not significantly different from that of healthy controls (SMD = 0.50, 95%CI = -1.10–2.10, *p* = 0.54, random-effects model, Fig. 13, and SMD = − 0.90, 95%CI = -2.13–0.52, *p* = 0.21, random-effects model, Fig. 14, Respectively).

### Subgroup analysis according to NOS score

Supplementary file 4 shows the forest plots of the Subgroup analysis according to the NOS score.

In the subgroup analysis based on the quality of studies, we found that patients with RAS had lower levels of SOD compared to healthy controls in high-quality studies (SMD = − 1.97, 95%CI = -3.17 to -0.77, *p* = 0.001), but not in moderate quality studies (SMD = 0.15, 95%CI = -0.77–1.07, *p* = 0.074, Supplementary file 4. Fig. S1).

In addition, it was shown that RAS patients had lower levels of GPx than healthy controls in moderate-quality studies (SMD = − 0.97, 95%CI = -1.24 to -0.70, *p* < 0.001) but not in high-quality studies (SMD = − 2.48, 95%CI = -5.58–0.61, *p* = 0.011, Supplementary file 4. Fig. S2).

We also found that there was no difference in CAT level between RAS patients and healthy controls in either moderate-quality studies (SMD = − 0.13, 95%CI = -1.26–1.01, *p* = 0.82) or high-quality studies (SMD = − 1.31, 95%CI = -2.72–0.09, *p* = 0.06, Supplementary file 4. Fig. S3).

Also, lower levels of serum TAS were found in RAS patients than healthy controls in either moderate-quality studies (SMD = − 0.80, 95%CI = -1.47 to -0.13, *p* = 0.02) or high-quality studies (SMD = − 1.39, 95%CI = -2.53 to -0.26, *p* = 0.01, Supplementary file 4. Fig. S4).

We showed that there was no difference in CAT level between patients with RAS and healthy controls in either moderate-quality studies (SMD = − 1.10, 95%CI = -0.48 to -0.29, *p* = 0.62) or high-quality studies (SMD = − 0.15, 95%CI = -0.60–0.29, *p* = 0.49, Supplementary file 4. Fig. S5).

Higher levels of serum MDA and TOS were found in patients with RAS in comparison to healthy controls in either moderate quality studies (SMD = 1.58, 95%CI = 0.87–2.29, *p* < 0.001, and SMD = 0.79, 95%CI = 0.17–1.40, *p* = 0.01, respectively), or high-quality studies (SMD = 2.96, 95%CI = 1.65–4.27, *p* < 0.001, and SMD = 4.63, 95%CI = 3.79–5.48, *p* < 0.001, Supplementary file 4. Fig. S6 and S7, respectively).

Accordingly, it was shown that RAS patients had lower levels of serum OSI than healthy controls in high-quality studies (SMD = 2.72, 95%CI = 2.11–3.33, *p* < 0.001) but not in moderate-quality studies (SMD = 0.88, 95%CI = -0.00–1.76, *p* = 0.05, Supplementary file 4. Fig. S8).

Also, it was shown that patients with RAS had higher levels of salivary MDA compared to healthy controls in high-quality studies (SMD = 1.52, 95%CI = 0.85–2.19, *p* < 0.001) but not in moderate-quality studies (SMD = − 0.70, 95%CI = -3.61–2.21, *p* = 0.47, Supplementary file 4. Fig. S9).

It was shown that patients with RAS had lower levels of serum Vit E compared to healthy controls in high-quality studies (SMD = − 0.64, 95%CI = -1.16 to -0.12, p = 0.01) but not in moderate-quality studies (SMD = 0.42, 95%CI = -2.73–3.56, *p* = 0.79, Supplementary file 4. Fig. S10).

In addition, the subgroup analysis showed that there was no difference in GSH level between patients with RAS and healthy controls in either moderate-quality studies (SMD = 0.68, 95%CI = -2.11–3.47, *p* = 0.63) or high-quality studies (SMD = − 1.99, 95%CI = -4.24–0.52, *p* = 0.08, Supplementary file 4. Fig. S11).

### Subgroup analysis according to country

Supplementary file 4 shows the forest plots of the Subgroup analysis according to the country.

In the subgroup analysis based on country, we found that compared to healthy controls, the SOD level of patients with RAS was not different in Turkey (SMD = − 0.31, 95%CI = -3.17–1.21, *p* = 0.68) but was lower in other countries (SMD = − 1.52, 95%CI = − 2.38 to -0.67, *p* < 0.001, Supplementary file 4. Fig. S12).

In the subgroup analysis based on country, we found that patients with RAS had lower levels of GPx compared to healthy controls in studies conducted in Turkey (SMD = − 2.02, 95%CI = -3.47 to -0.56, *p* = 0.007) but not in other countries (SMD = − 1.83, 95%CI = -4.59–0.93, *p* = 0.19, Supplementary file 4. Fig. S13).

We also found that there was no difference in CAT level between RAS patients and healthy controls in studies conducted in either Turkey (SMD = − 1.78, 95%CI = -4.01–0.46, *p* = 0.12) or other countries (SMD = − 0.15, 95%CI = -0.94–0.65, *p* = 0.71, Supplementary file 4. Fig. S14).

Also, lower levels of serum TAS were found in RAS patients than healthy controls in studies conducted in either Turkey (SMD = − 0.91, 95%CI = -1.81to -0.01, *p* = 0.04) or other countries (SMD = − 1.06, 95%CI = -1.94 to -0.18, *p* = 0.01, Supplementary file 4. Fig. S15).

We also found that there was no difference in salivary TAS level between RAS patients and healthy controls in studies conducted in either Turkey (SMD = − 0.15, 95%CI = -0.64–0.34, *p* = 0.54) or other countries (SMD = − 0.12, 95%CI = -0.44–0.21, *p* = 0.48, Supplementary file 4. Fig. S16).

In addition, higher levels of serum MDA, TAS, and OSI were found in RAS patients than healthy controls in studies conducted in either Turkey (SMD = 1.79, 95%CI = 0.88–2.70, *p* < 0.001, and SMD = 1.77, 95%CI = 0.14–3.39, *p* = 0.03, and SMD = 1.39, 95%CI = 0.05–2.73, *p* = 0.04, respectively), or other countries (SMD = 2.49, 95%CI = 1.34–3.65, *p* < 0.001, and SMD = 0.67, 95%CI = 0.24–1.10, *p* = 0.002, and SMD = 0.72, 95%CI = 0.29–1.15, *p* = 0.001, Supplementary file 4. Fig. S17, S18, and S19, respectively).

In the subgroup analysis based on country, we found that patients with RAS had higher levels of salivary MDA compared to healthy controls in studies conducted in Turkey (SMD = 1.46, 95%CI = 0.82–2.09, *p* < 0.007) but not in other countries (SMD = 0.66, 95%CI = -0.64–1.97, *p* = 0.31, Supplementary file 4. Fig. S20).

In the subgroup analysis based on country, we found that patients with RAS had lower levels of Vit E compared to healthy controls in studies conducted in Turkey (SMD = − 0.64, 95%CI = -1.16–0.12, *p* = 0.01) but not in other countries (SMD = 0.42, 95%CI = -2.73–3.56, *p* = 0.79, Supplementary file 4. Fig. S21).

We also found that there was no difference in GSH level between RAS patients and healthy controls in studies conducted in either Turkey (SMD = 0.68, 95%CI = -2.11–3.47, *p* = 0.63) or other countries (SMD = − 1.99, 95%CI = -4.24–0.26, *p* = 0.08, Supplementary file 4. Fig. S22).

### Sensitivity analysis

As seen in Supplementary File 5, the pooled estimates did not change after the deletion of each study in sensitivity analysis, showing that our results were stable.

### Meta-regression analysis

In the meta-regression analysis, there was no significant effect of the mean age of cases (B = 0.12, *p* = 0.44) and percentage of patients with minor RAS (B = − 0.05, *p* = 0.34) on SOD. However, gender had a significant effect (B = 0.002, *p* = 0.04) on the association between SOD and RAS, so it could be the source of heterogeneity among studies on this biomarker.

In the meta-regression analysis of GPx, there was no significant effect of gender (B = − 0.05, *p* = 0.71) and mean age of cases (B = 0.02, *p* = 0.07) on GPx. However, the percentage of patients with minor RAS had a significant effect (B = − 0.16, *p* = 0.03) on the association between GPx and RAS; so it could be the source of heterogeneity.

Also, the meta-regression analysis of CAT showed no significant effect of gender (B = 0.13, *p* = 0.19) and mean age of cases (B = − 0.10, *p* = 0.72), and percentage of patients with minor RAS (B = − 0.08, *p* = 0.38) on CAT; so they could not be the source of heterogeneity.

In addition, we found no significant effect of the mean age of cases (B = 0.05, *p* = 0.81) percentage of patients with minor RAS (B = − 0.02, *p* = 0.42) on serum TAS. However, gender significantly affected the association between serum TAS and RAS (B = − 0.07, *p* = 0.002).

We could not find the source of heterogeneity among studies on salivary TAS because there was no significant effect of gender (B = − 0.01, *p* = 0.35) and mean age of cases (B = − 0.0002, *p* = 0.99) percentage of patients with minor RAS (B = 0.004, *p* = 0.66) on salivary TAS.

Similarly, we could not identify the source of heterogeneity among studies on serum MDA because there was no significant effect of gender (B = 0.0009, *p* = 0.97), mean age of cases (B = − 0.01, *p* = 0.86), and percentage of patients with minor RAS (B = 0.01, *p* = 0.72) on serum MDA.

The source of heterogeneity was not identified in the meta-regression analysis since there was no significant effect of gender (B = 0.11, *p* = 0.33) and mean age of cases (B = − 0.41, *p* = 0.49) on serum TOS.

Similarly, the source of heterogeneity was not identified in the meta-regression analysis since there was no significant effect of gender (B = 0.10, *p* = 0.02) and mean age of cases (B = 0.06, *p* = 0.89) on serum OSI.

According to meta-regression analysis, the mean age of cases could be the source of heterogeneity (B = 0.06, *p* < 0.001). However, gender (B = 0.07, *p* = 0.17) and percentage of patients with minor RAS (B = 0.004, *p* = 0.92) had no significant effect on salivary MDA.

According to meta-regression analysis, gender could be the source of heterogeneity (B = − 0.11, *p* < 0.001). However, the mean age of cases (B = − 0.19, *p* = 0.37) had no significant effect on Vit E.

In the case of Vit C, gender was shown as a possible source of heterogeneity (B = − 1.20, *p* < 0.001). However, the mean age of cases (B = − 4.77, *p* = 0.26) had no significant effect on Vit C.

In the case of salivary UA, gender (B = 0.06, *p* = 0.71) and mean age of cases (B = − 0.09, *p* = 0.69) had no significant effect on this biomarker.

Finally, the mean age of cases (B = − 0.04, *p* < 0.001) and percentage of patients with minor RAS (B = 0.16, *p* = 0.01) could be the sources of heterogeneity among studies on GSH; however, gender (B = 0.14, *p* = 0.48) had not any significant effect on this biomarker.

### Publication bias

As seen in funnel plot shown in Supplementary file 6, there was no publication bias among studies on erythrocyte SOD and GPx activity (Egger’s test *p* = 0.46, and 0.30, Respectively), erythrocyte CAT (Egger’s test *p* = 0.66), serum TAS level (Egger’s test *p* = 0.19), salivary TAS level(Egger’s test *p* = 0.74) serum OSI (Egger’s test *p* = 0.17), salivary MDA (Egger’s test p = 0.66), serum Vit E and C (Egger’s test *p* = 0.24 and 0.72, respectively), serum UA (Egger’s test *p* = 0.47) and serum GSH (Egger’s test *p* = 0.78). However, there was me evidence of publication bias among studies on serum MDA and TOS level (Egger’s test *p* = 0.001 and 0.04, Respectively).

## Discussion

Our study aimed to clarify and quantify the oxidative stress and antioxidant markers in the saliva and serum/plasma of RAS. The reviewed data and the results of our meta-analyses point to a role for significantly increased oxidative stress indicators (TOS, OSI, MDA) and decreased antioxidant markers (TOS, erythrocyte SOD and GPx activity) in individuals with RAS compared to healthy controls.

Young adults are at a high risk for developing RAS, which must be distinguished from other recurrent ulceration-causing conditions such as gluten-sensitive enteropathy, Behçet disease, hematinic deficiencies, inflammatory bowel diseases, and other syndrome s[5]. The nonkeratinized mucosa, notably the labial and buccal mucosa and tongue, is where lesions of RAS are most often discovered. The extensively keratinized mucosa of the gum and palate participate less often [46]. RAS affects around 25% of the global population; however, the incidence fluctuates between 5 and 50% depending on ethnic and socioeconomic factors(2). The cause of RAS lesions is unknown; however, numerous local (trauma), systemic, immunological, genetic, dietary, microbial, and allergic variables have been hypothesized [47–53].

Additionally, immunosuppressive medications like the mammalian target of rapamycin protein kinase inhibitors and calcineurin have been linked to severe aphthous stomatitis [54]. All of these difficulties may disrupt the organism’s oxidant-antioxidant balance, creating free radicals [55–58]. An oxidative stress condition may therefore damage the immune system. It is caused by an increase in free radicals and may damage cells. Cells contain antioxidant systems that include enzymes like catalase, superoxide dismutase, and GPx to preserve themselves from oxidative stress. Non-enzymatic antioxidants include vitamins A, C, and E, reduced glutathione (GSH), UA, and melatonin [59–62].

An imbalance between prooxidant chemicals (like reactive nitrogen species (RNS) and ROS) and the antioxidant system’s ability (non-enzymatic and enzymatic antioxidants) results in oxidative stress. ROS are produced from a variety of exogenous and endogenous sources. The oral cavity is a crucial location for exogenous sources of ROS. Exogenous causes of oxidative stress are oral tissue exposure to microbial, chemical, and thermal stimuli. Additionally, several behavioral variables increase the synthesis of exogenous ROS (alcohol use, smoking, and chewing betel nuts) [38]. Endogenous sources relate to acute or chronic oral infections, such as RAS and periodontitis. Significant levels of ROS are known to be produced by inflammatory cells, and ROS then increases the inflammatory response [36]. Multiple pathways, such as DNA damage, protein oxidation, and lipid peroxidation (LPO) damage, are used by ROS to induce oxidative damage to tissues, leading to disorders like RAS [19].

Our meta-analysis revealed that oxidative stress and antioxidant indicators might be viable biomarkers for RAS diagnosis [15–44]. A rise in oxidants and a reduction in antioxidants might signify aphthous incidence.

Because the oral cavity represents the beginning of the digestive system, saliva, which contains numerous antioxidants like albumin, UA, and ascorbic acid, serves as the first line of protection against OS. Ergun et al. found a significant association between oxidant and antioxidant serum and salivary levels [38]. These findings imply that saliva is viable for evaluating oxidative stress levels, allowing for non-invasive RAS diagnosis. Saliva may function as a diagnostic fluid and has numerous benefits over urine and blood samples: It is safe, non-invasive, painless, and simple to collect. Furthermore, salivary OS biomarkers indicate the condition of local oral oxidative stress, which may more accurately reflect the actual state of the local oral microenvironment. In contrast to these findings, we found that the markers in saliva and blood had not the same variation tendency. Patients with RAS had significantly higher serum MDA and lower TAS compared to healthy controls, however, the salivary levels of these biomarkers were not different between two groups. It could be due to the limited number of studies reporting salivary biomarkers. So, using saliva to evaluate oxidative stress in RAS needs to be more studied. There are currently no uniform and established procedures for collecting saliva, and it is still unknown whether saliva has to be centrifuged or stimulated after sampling, how long it should be stored at what temperature, or how to do analysis.

### Limitations and strengths

Our study had several strengths: 1) we conducted a comprehensive and extensive search to discover all publications on the relationship of RAS with antioxidant biomarker and oxidative stress. 2) we rigorously assessed and analyzed the included studies one-by-one. 3) In order to derive comprehensive conclusions, we processed the data through quantitative synthesis. Yet, by analyzing the heterogeneity of various investigations, it may be possible to make a fairly impartial assessment of oxidative stress and antioxidant indicators in RAS.

There are some limitations to this meta-analysis. First, most published research has compared oxidant-antioxidant state in RAS patients to healthy humans. However, the diagnostic value of salivary and serum redox biomarkers in RAS diagnosis has not been well established. When employing biomarkers as diagnostic/prognostic indicators in RAS, there has been no documented estimation of specificity, sensitivity, cluster analysis, or ROC analysis, predictive values, etc. More research in this area will aid in the identification of a valid and straightforward diagnostic or prognostic indicator among the antioxidant and OS markers that might be exploited as a treatment target in clinical practice. Furthermore, we discovered the heterogeneity across the included publications. The substantial heterogeneity in the results could be explained in part by variations in mean age, male percentage, percentage of patients with minor RAS, as seen in meta-regression analysis.

## Conclusion

The relationship between oxidative stress and RAS is well established in this meta-analysis. Although the molecular processes underlying the etiology of this pathology remain unknown, evidence indicating oxidative stress has a significant role in the pathogenesis of RAS has been revealed. This hypothesis is supported by a rise in free radicals in patients’ saliva and oral tissues, as well as a decrease in the activity of antioxidant defense mechanisms. Based on these considerations, more research in this area will make it evident if the onset of oxidative stress causes or is a predisposing factor in this disease. Future research may lead to identifying a specific and reliable diagnostic marker among the many radical molecules or components of the antioxidant barrier that would then be exploited as a therapeutic target in clinical practice.

### Supplementary Information

**Additional file 1.**

**Additional file 2.**

**Additional file 3.**

**Additional file 4.**

**Additional file 5.**

**Additional file 6.**

## Data Availability

The dataset supporting the conclusions of this article is included within the article.

## Abbreviations

SMD: Standardized mean difference
95% CI: 95% confidence interval
N: Number
NOS: The Newcastle-Ottawa Quality Assessment Scale
R: Retrospective
P: Prospective
PRISMA: Preferred Reporting Items for Systematic Reviews and Meta-Analyses
RAS: Recurrent aphthous stomatitis
ROS: Reactive oxygen species
SOD: Superoxide dismutase
CAT: Catalase
TOS: Total oxidant status
UA: Uric acid
Vit E: Vitamin E
Vit C: Vitamin C
MDA: Malondialdehyde
OSI: Oxidative stress index
GSH: Reduced glutathione
RNS: Reactive nitrogen species
LPO: Lipid peroxidation
GPx: Glutathione peroxidase
TAS: Total antioxidant status
