# Supplementary material for: Systematic review and meta-analysis of oxidative stress and antioxidant markers in recurrent aphthous stomatitis
Source: BMC Oral Health. 2023 Dec 2;23:960. doi: 10.1186/s12903-023-03636-1 (PMC10693709; doi:10.1186/s12903-023-03636-1)
Supplement: Supplementary file 3 — Additional file 3. [file 12903_2023_3636_MOESM3_ESM.docx]

1. **Results of sensitivity analysis of differences in erythrocyte superoxide dismutase activity between patients with recurrent aphthous stomatitis and healthy controls**

------------------------------------------------------------------------------

Study omitted | Estimate [95% Conf. Interval]

-------------------+----------------------------------------------------------

Cimen (2003) | -1.1844585 -2.020597 -.34832016

Altinyazar (2006) | -1.2440072 -2.0288775 -.45913696

Momen (2010) | -1.0512707 -1.9517205 -.15082084

Ozturn (2013) | -.76237941 -1.5255008 .00074192

Gupta (2014) | -.61874676 -1.3021286 .06463514

Jesica (2017) | -1.0730863 -1.9890561 -.15711641

Sebsa (2020) | -.91742527 -1.7721021 -.06274833

Zhang (2017) | -1.0259402 -2.0169795 -.03490095

Gunduz (2004) | -1.1504058 -2.0129826 -.28782886

-------------------+----------------------------------------------------------

Combined | -1.0008886 -1.7887928 -.2129844

1. **Results of sensitivity analysis of differences in erythrocyte Glutathione peroxidase activity between patients with recurrent aphthous stomatitis and healthy controls**

------------------------------------------------------------------------------

Study omitted | Estimate [95% Conf. Interval]

-------------------+----------------------------------------------------------

Arikan (2009) | -1.8728372 -3.6450284 -.10064601

Cimen (2003) | -2.1539931 -4.0419569 -.26602918

Ozturn (2013) | -1.6300883 -3.263864 .00368746

Gupta (2014) | -1.0978973 -2.4870937 .2912991

Jesica (2017) | -2.5749354 -3.93187 -1.2180007

Zhang (2017) | -2.1191528 -4.3806005 .14229478

-------------------+----------------------------------------------------------

Combined | -1.9026674 -3.428209 -.37712583

------------------------------------------------------------------------------

1. **Results of sensitivity analysis of differences in erythrocyte Catalase activity between patients with recurrent aphthous stomatitis and healthy controls**

------------------------------------------------------------------------------

Study omitted | Estimate [95% Conf. Interval]

-------------------+----------------------------------------------------------

Cimen (2003) | -.74045444 -1.6907798 .20987093

Altinyazar (2006) | -.92115062 -1.8259529 -.01634835

Momen (2010) | -.81747282 -1.7890295 .15408388

Ozturn (2013) | .01363425 -.60677284 .63404131

Gupta (2014) | -.91652662 -1.825449 -.00760426

Jesica (2017) | -.8448211 -1.8262206 .13657846

Sebsa (2020) | -.79117769 -1.7616377 .17928231

Zhang (2017) | -.62087333 -1.5186793 .27693257

Gunduz (2004) | -.88011771 -1.819134 .05889852

-------------------+----------------------------------------------------------

Combined | -.70810773 -1.5607053 .14448986

1. **Results of sensitivity analysis of differences in** **serum total antioxidant status level between patients with recurrent aphthous stomatitis and healthy controls**

------------------------------------------------------------------------------

Study omitted | Estimate [95% Conf. Interval]

-------------------+----------------------------------------------------------

Akoglu (2013) | -.86394471 -1.4568974 -.27099204

Bilgili (2013) | -1.0248857 -1.6798478 -.36992347

Avci (2014) | -1.0615994 -1.7045044 -.41869426

Momen (2010) | -1.0654047 -1.7132778 -.4175314

Ekinci (2019) | -1.1225467 -1.7328924 -.51220095

Turgul (2016) | -.87440902 -1.4749345 -.27388364

Al-Essa (2013) | -1.0504128 -1.7011564 -.39966911

Zhang (2018) | -1.0225776 -1.731704 -.31345144

Hussein (2016) | -.67473674 -1.1394012 -.2100722

Kurku (2022) | -1.0792249 -1.7294644 -.42898545

-------------------+----------------------------------------------------------

Combined | -.9817141 -1.5704035 -.39302468

1. **Results of sensitivity analysis of differences in** **salivary total antioxidant status level between patients with recurrent aphthous stomatitis and healthy controls**

------------------------------------------------------------------------------

Study omitted | Estimate [95% Conf. Interval]

-------------------+----------------------------------------------------------

Babaee (2016) | -.03528951 -.26476279 .19418378

Caglayan (2008) | -.11637743 -.44068497 .20793013

Azizi (2012) | -.197358 -.45416552 .05944952

Momen (2010) | -.14878422 -.46610719 .16853875

Al-Essa (2013) | -.06858515 -.36256793 .22539763

Rezaei (2018) | -.17414625 -.46912175 .12082923

-------------------+----------------------------------------------------------

Combined | -.12326185 -.38498741 .13846371

-----------------------------------------------------------------------------

1. **Results of sensitivity analysis of differences in** **serum Malondialdehyde between patients with recurrent aphthous stomatitis and healthy controls**

------------------------------------------------------------------------------

Study omitted | Estimate [95% Conf. Interval]

-------------------+----------------------------------------------------------

Arikan (2009) | 2.1669331 1.4289824 2.9048836

Cimen (2003) | 2.209069 1.4716737 2.9464645

Avci (2014) | 2.2280006 1.4921315 2.96387

Khademi (2017) | 1.99467 1.3104118 2.6789281

Altinyazar (2006) | 2.2029028 1.4629295 2.9428759

Saral (2005) | 2.0410514 1.3387324 2.7433705

Ozturn (2013) | 1.8674586 1.2473563 2.487561

Al-Essa (2013) | 2.2202954 1.4761066 2.9644842

Li (2016) | 2.23088 1.4537784 3.0079818

Sebsa (2020) | 2.1701798 1.4228352 2.9175241

Bagan (2014) | 1.94598 1.2858366 2.6061232

Hussein (2016) | 1.9037945 1.2695487 2.5380404

Yardim (2006) | 2.2367976 1.5079471 2.9656482

-------------------+----------------------------------------------------------

Combined | 2.1083155 1.4272464 2.7893847

------------------------------------------------------------------------------

1. **Results of sensitivity analysis of differences in** **serum total oxidant status level between patients with recurrent aphthous stomatitis and healthy controls**

------------------------------------------------------------------------------

Study omitted | Estimate [95% Conf. Interval]

-------------------+----------------------------------------------------------

Bilgili (2013) | 1.4629632 .01213206 2.9137945

Avci (2014) | 1.8240455 .33263919 3.3154519

Ekinci (2019) | 1.8510368 .34268689 3.3593867

Turgul (2016) | .78931141 .1739898 1.4046329

Kurku (2022) | 1.76799 .14403762 3.3919423

-------------------+----------------------------------------------------------

Combined | 1.531326 .33982471 2.7228272

------------------------------------------------------------------------------

1. **Results of sensitivity analysis of differences in** **serum oxidative stress index level between patients with recurrent aphthous stomatitis and healthy controls**

------------------------------------------------------------------------------

Study omitted | Estimate [95% Conf. Interval]

-------------------+----------------------------------------------------------

Bilgili (2013) | .95261222 -.07622191 1.9814464

Avci (2014) | 1.5295444 .36722457 2.6918643

Ekinci (2019) | 1.4997199 .29361886 2.7058208

Turgul (2016) | .87896246 -.00136752 1.7592924

Kurku (2022) | 1.3896917 .04710121 2.7322822

-------------------+----------------------------------------------------------

Combined | 1.2493021 .25308686 2.2455173

------------------------------------------------------------------------------

1. **Results of sensitivity analysis of differences in salivary Malondialdehyde level between patients with recurrent aphthous stomatitis and healthy controls**

------------------------------------------------------------------------------

Study omitted | Estimate [95% Conf. Interval]

-------------------+----------------------------------------------------------

Babaee (2016) | .76181895 -.60536575 2.1290038

Ziaudeen (2001) | .72548157 -.63810199 2.0890651

Khademi (2017) | 1.3615283 .78525645 1.9378002

Saral (2005) | .66480845 -.63868123 1.9682981

Al-Essa (2013) | .80055094 -.58338541 2.1844873

Sebsa (2020) | .44405001 -.66900527 1.5571053

-------------------+----------------------------------------------------------

Combined | .79727446 -.29876768 1.8933166

------------------------------------------------------------------------------

1. **Results of sensitivity analysis of differences in serum vitamin E level between patients with recurrent aphthous stomatitis and healthy controls**

------------------------------------------------------------------------------

Study omitted | Estimate [95% Conf. Interval]

-------------------+----------------------------------------------------------

Arikan (2009) | -.0353115 -1.7946481 1.724025

Khademi (2017) | -.8680135 -1.3403053 -.39572173

Saral (2005) | .1402867 -1.6130517 1.893625

Li (2016) | .24115156 -1.4349995 1.9173026

-------------------+----------------------------------------------------------

Combined | -.12746338 -1.3840066 1.1290799

------------------------------------------------------------------------------

1. **Results of sensitivity analysis of differences in serum vitamin C level between patients with recurrent aphthous stomatitis and healthy controls**

------------------------------------------------------------------------------

Study omitted | Estimate [95% Conf. Interval]

-------------------+----------------------------------------------------------

Khademi (2017) | -.51880139 -.88219404 -.15540883

Saral (2005) | .27470025 -1.0960534 1.6454538

Li (2016) | .0957054 -1.6754962 1.866907

-------------------+----------------------------------------------------------

Combined | -.08233558 -.99238306 .8277119

------------------------------------------------------------------------------

1. **Results of sensitivity analysis of differences in salivary uric acid level between patients with recurrent aphthous stomatitis and healthy controls**

’

-----------------------------------------------------------------------------

Study omitted | Estimate [95% Conf. Interval]

-------------------+----------------------------------------------------------

Ziaudeen (2001) | 1.3138282 .81998342 1.8076731

Jesica (2017) | -.03664485 -2.1691141 2.0958245

Sebsa (2020) | .21747634 -2.40927 2.8442228

-------------------+----------------------------------------------------------

Combined | .49609989 -1.1044755 2.0966752

------------------------------------------------------------------------------

1. **Results of sensitivity analysis of differences in** **serum reduced glutathione level between patients with recurrent aphthous stomatitis and healthy controls**

------------------------------------------------------------------------------

Study omitted | Estimate [95% Conf. Interval]

-------------------+----------------------------------------------------------

Avci (2014) | -1.6144725 -2.9058342 -.32311094

Momen (2010) | -1.1642921 -3.0517485 .7231642

Sebsa (2020) | -.7829963 -2.5689292 1.0029366

Bagan (2014) | -.00071636 -1.1784159 1.1769831

Zhang (2022) | -.96735454 -3.1531415 1.2184325

-------------------+----------------------------------------------------------

Combined | -.89623567 -2.3092152 .51674385

------------------------------------------------------------------------------
