# Supplementary material for: Systematic review and meta-analysis of oxidative stress and antioxidant markers in recurrent aphthous stomatitis
Source: BMC Oral Health. 2023 Dec 2;23:960. doi: 10.1186/s12903-023-03636-1 (PMC10693709; doi:10.1186/s12903-023-03636-1)
Supplement: Supplementary file 4 — Additional file 4. [file 12903_2023_3636_MOESM4_ESM.docx]

**Title**

Systematic review and meta-analysis of oxidative stress and antioxidant markers in recurrent aphthous stomatitis

**Funnel plots assessing publication bias**
