# Supplementary material for: Systematic review and meta-analysis of oxidative stress and antioxidant markers in recurrent aphthous stomatitis
Source: BMC Oral Health. 2023 Dec 2;23:960. doi: 10.1186/s12903-023-03636-1 (PMC10693709; doi:10.1186/s12903-023-03636-1)
Supplement: Supplementary file 5 — Additional file 5. [file 12903_2023_3636_MOESM5_ESM.docx]

**Subgroup analysis according to NOS score and country in which the study was conducted**

**Figure S1.** Subgroup analysis of differences in erythrocyte superoxide dismutase activity between patients with recurrent aphthous stomatitis and healthy controls according to NOS score

**Figure S2.** Subgroup analysis of differences in erythrocyte Glutathione peroxidase activity between patients with recurrent aphthous stomatitis and healthy controls according to NOS score

**Figure S3.** Subgroup analysis of differences in erythrocyte Catalase activity between patients with recurrent aphthous stomatitis and healthy controls according to NOS score

**Figure S4.** Subgroup analysis of differences in serum total antioxidant status level between patients with recurrent aphthous stomatitis and healthy controls according to NOS score

**Figure S5.** Subgroup analysis of differences in salivary total antioxidant status between patients with recurrent aphthous stomatitis and healthy controls according to NOS score

**Figure S6.** Subgroup analysis of differences in serum Malondialdehyde between patients with recurrent aphthous stomatitis and healthy controls according to NOS score

**Figure S7.** Subgroup analysis of differences in serum total oxidant status level between patients with recurrent aphthous stomatitis and healthy controls according to NOS score

**Figure S8.** Subgroup analysis of differences in serum oxidative stress index level between patients with recurrent aphthous stomatitis and healthy controls according to NOS score

**Figure S9.** Subgroup analysis of differences in salivary Malondialdehyde level between patients with recurrent aphthous stomatitis and healthy controls according to NOS score

**Figure S10.** Subgroup analysis of differences in serum vitamin E level between patients with recurrent aphthous stomatitis and healthy controls according to NOS score

**Figure S11.** Subgroup analysis of differences in serum reduced glutathione level between patients with recurrent aphthous stomatitis and healthy controls according to NOS score

**Figure S12.** Subgroup analysis of differences in erythrocyte superoxide dismutase activity between patients with recurrent aphthous stomatitis and healthy controls according to the country in which the study was conducted

**Figure S13.** Subgroup analysis of differences in erythrocyte Glutathione peroxidase activity between patients with recurrent aphthous stomatitis and healthy controls according to the country in which the study was conducted

**Figure S14.** Subgroup analysis of differences in erythrocyte Catalase activity between patients with recurrent aphthous stomatitis and healthy controls according to the country in which the study was conducted

 **Figure S15.** Subgroup analysis of differences in serum total antioxidant status level between patients with recurrent aphthous stomatitis and healthy controls according to the country in which the study was conducted

**Figure S16.** Subgroup analysis of differences in salivary total antioxidant status between patients with recurrent aphthous stomatitis and healthy controls according to the country in which the study was conducted

**Figure S17.** Subgroup analysis of differences in serum Malondialdehyde between patients with recurrent aphthous stomatitis and healthy controls according to the country in which the study was conducted

**Figure S18.** Subgroup analysis of differences in serum total oxidant status level between patients with recurrent aphthous stomatitis and healthy controls according to the country in which the study was conducted

**Figure S19.** Subgroup analysis of differences in serum oxidative stress index level between patients with recurrent aphthous stomatitis and healthy controls according to the country in which the study was conducted

**Figure S20.** Subgroup analysis of differences in salivary Malondialdehyde level between patients with recurrent aphthous stomatitis and healthy controls according to the country in which the study was conducted

**Figure S21.** Subgroup analysis of differences in serum vitamin E level between patients with recurrent aphthous stomatitis and healthy controls according to the country in which the study was conducted

**Figure S22.** Subgroup analysis of differences in serum reduced glutathione level between patients with recurrent aphthous stomatitis and healthy controls according to the country in which the study was conducted
