# Supplementary material for: Systematic review and meta-analysis of oxidative stress and antioxidant markers in recurrent aphthous stomatitis
Source: BMC Oral Health. 2023 Dec 2;23:960. doi: 10.1186/s12903-023-03636-1 (PMC10693709; doi:10.1186/s12903-023-03636-1)
Supplement: Supplementary file 6 — Additional file 6. [file 12903_2023_3636_MOESM6_ESM.docx]

| **Supplementary file 3.** Quality assessment of included studies using NOS questionnaire | | | | | | | | |
| --- | --- | --- | --- | --- | --- | --- | --- | --- |
| Author | Is the case definition adequate? | Representativeness of the cases | Selection of Controls | Definition of Controls | Comparability of cases and controls on the basis of the design or analysis | Ascertainment of exposure | Same method of ascertainment for cases and controls | Non-Response Rate |
| Arikan,2009 | ● | ● | _ | ● | ●● | ● | ● | ● |
| Cimen, 2003 | ● | ● | _ | ● | ● | ● | ● | ● |
| Babaee, 2016 | ● | ● | _ | ● | ●● | ● | ● | ● |
| Akoglu, 2013 | ● | ● | ● | ● | ● | ● | ● | ● |
| Ziaudeen, 2001 | ● | ● | ● | ● | ● | ● | ● | ● |
| Bilgili, 2013 | ● | ● | _ | ● | ● | ● | ● | ● |
| Avci, 2014 | ● | ● | _ | ● | ● | ● | ● | ● |
| Khademi, 2014 | ● | ● | _ | _ | ● | ● | ● | ● |
| Altinyazar, 2006 | ● | ● | _ | ● | ● | ● | ● | ● |
| Caglayan , 2008 | ● | ● | _ | _ | ● | ● | ● | ● |
| Azizi, 2012 | ● | ● | _ | _ | ● | ● | ● | ● |
| Saral, 2005 | ● | ● | ● | ● | ●● | ● | ● | ● |
| Momen, 2010 | ● | ● | ● | ● | ● | ● | ● | ● |
| Ozturk, 2013 | ● | ● | _ | ● | ●● | ● | ● | ● |
| Gupta, 2014 | ● | ● | ● | ● | ● | ● | ● | ● |
| Ekinci, 2019 | ● | ● |  | ● | ● | ● | ● | ● |
| Turgul, 2016 | ● | ● | ● | ● | ● | ● | ● | ● |
| Al-Essa, 2013 | ● | ● | _ | _ | ● | ● | ● | ● |
| Li, 2016 | ● | ● | ● | _ | ● | ● | ● | ● |
| Jesija, 2017 | ● | ● | ● | ● | ● | ● | ● | ● |
| Zhang, 2018 | ● | ● | _ | ● | ● | ● | ● | ● |
| Sebea, 2020 | ● | ● | ● | ● | ● | ● | ● | ● |
| Rezaei, 2018 | ● | ● | ● | ● | ●● | ● | ● | ● |
| Bagan, 2014 | ● | ● | _ | ● | ●● | ● | ● | ● |
| Zhang, 2017 | ● | ● | ● | _ | ● | ● | ● | ● |
| Hussein, 2016 | ● | ● | ● | _ | ● | ● | ● | ● |
| Gunduz, 2004 | ● | ● | ● | _ | ● | ● | ● | ● |
| Yardim, 2006 | ● | ● | _ | _ | ● | ● | ● | ● |
| Kurku, 2022 | ● | ● | ● | _ | ● | ● | ● | ● |
| Zhang, 2022 | ● | ● | ● | _ | ● | ● | ● | ● |
